# Breast Cancer: An Examination of the Potential of ACKR3 to Modify the Response of CXCR4 to CXCL12

**DOI:** 10.3390/ijms19113592

**Published:** 2018-11-14

**Authors:** Irene del Molino del Barrio, Georgina C. Wilkins, Annette Meeson, Simi Ali, John A. Kirby

**Affiliations:** 1 Applied Immunobiology and Transplantation Group, Institute of Cellular Medicine, Medical School, University of Newcastle Upon Tyne, Newcastle upon Tyne NE2 4HH, UK; irene.delmolinodelbarrio@dpag.ox.ac.uk (I.d.M.d.B.); G.Wilkins2@newcastle.ac.uk (G.C.W.); J.A.Kirby@newcastle.ac.uk (J.A.K.); 2 Institute of Genetic Medicine, International Centre for Life, University of Newcastle Upon Tyne, Newcastle upon Tyne NE1 3BZ, UK; annette.meeson@newcastle.ac.uk

**Keywords:** chemokines, metastasis, heterodimerization, CXCR4, ACKR3, CXCR7

## Abstract

Upon binding with the chemokine CXCL12, the chemokine receptor CXCR4 has been shown to promote breast cancer progression. This process, however, can be affected by the expression of the atypical chemokine receptor ACKR3. Given ACKR3’s ability to form heterodimers with CXCR4, we investigated how dual expression of both receptors differed from their lone expression in terms of their signalling pathways. We created single and double CXCR4 and/or ACKR3 Chinese hamster ovary (CHO) cell transfectants. ERK and Akt phosphorylation after CXCL12 stimulation was assessed and correlated with receptor internalization. Functional consequences in cell migration and proliferation were determined through wound healing assays and calcium flux. Initial experiments showed that CXCR4 and ACKR3 were upregulated in primary breast cancer and that CXCR4 and ACKR3 could form heterodimers in transfected CHO cells. This co-expression modified CXCR4’s Akt activation after CXCL12’s stimulation but not ERK phosphorylation (*p* < 0.05). To assess this signalling disparity, receptor internalization was assessed and it was observed that ACKR3 was recycled to the surface whilst CXCR4 was degraded (*p* < 0.01), a process that could be partially inhibited with a proteasome inhibitor (*p* < 0.01). Internalization was also assessed with the ACKR3 agonist VUF11207, which caused both CXCR4 and ACKR3 to be degraded after internalization (*p* < 0.05 and *p* < 0.001), highlighting its potential as a dual targeting drug. Interestingly, we observed that CXCR4 but not ACKR3, activated calcium flux after CXCL12 stimulation (*p* < 0.05) and its co-expression could increase cellular migration (*p* < 0.01). These findings suggest that both receptors can signal through ERK and Akt pathways but co-expression can alter their kinetics and internalization pathways.

## 1. Introduction

Breast cancer is the most prevalent cancer in the United States despite being predominantly a female disease, with 246,660 new cases expected to occur in 2016 [1]. Although there has been a decline in mortality rates thanks to improvements in targeted treatments and prevention, breast cancer is still expected to be the second most common cause of cancer death due to its metastasis to vital organs [2,3,4]. Thus, it remains vital to develop new targeted therapies to prevent this spread.

Since their discovery over 30 years ago, numerous chemokines have been identified in relation to cancer spread. Chemokines, or chemotactic cytokines are small (8–14 kDa) proteins that bind to G-protein-coupled chemokine receptors (GPCR) in order to induce chemotaxis [5]. Upon binding, chemokines can activate multiple signalling pathways and gene expression but it was their role in promoting leukocyte migration to the site of inflammation that allowed for the finding of the link between chemokines and cancer metastasis [6]. The discovery that cancer cells overexpress the chemokine receptor CXCR4 and that its blocking can inhibit metastasis to organs that express its ligand, the chemokine CXCL12 [7,8,9], opened new therapeutic avenues for the targeting of metastatic breast cancer.

Furthermore, it was recently discovered that the atypical chemokine receptor ACKR3 is also upregulated in many cancers, including breast [10,11,12]; however, reports are contradictory on whether ACKR3 expression is pro- or anti-metastatic. Unlike other chemokine receptors, ACKR3 has not been shown to activate G-proteins, most likely due to a change in the alanine and valine in the DRYLAIV motif that may affect the potential coupling [13]. This means that upon the binding of its ligands CXCL11 and CXCL12, ACKR3 does not activate G-proteins and instead it mediates their capture and degradation via β-arrestin due to its tenfold higher affinity for CXCL12 than CXCR4 [14]. However, it was recently proposed that β-arrestin can serve as a scaffold to which several kinases can bind and become activated [15], including ERK [15,16] and Akt [17].

Adding further complexity, ACKR3 can form heterodimers with CXCR4 as efficiently as homodimers [18,19,20] but its functional consequences are still contradictory. Indeed, there is compelling evidence that these complexes can have distinct effects from their corresponding homodimers [21], possibly through differential recruitment. It has been proposed that the formation of this heterodimer causes conformational changes that affect the CXCR4/G_αi_ interaction, which is reflected in an altered signalling response [20]. This was further explored by Décaillot and colleagues, who suggested that heterodimerization caused the abrogation of the G-protein in favour of β-arrestin signalling [22]. Heterodimerization, thus, adds a new layer of intricacy to receptor-ligand interactions but also opens exciting possibilities for a more precise targeting strategy.

The current study was designed to explore how the role of CXCR4/ACKR3 co-expression differs from their homodimer counterparts in a CHO model. In this study we show that ACKR3 activates kinases differently to CXCR4 and modulates ERK and Akt activation span when co-expressed. We demonstrate that co-expression can also alter receptor internalization, in particular when cross-desensitizing with the ACKR3 agonist VUF11207. We also describe that, although ACKR3 does not directly mediate migration towards CXCL12, it can enhance migration of cells co-expressing CXCR4 and ACKR3 as compared to CXCR4 alone. However, this mechanism does not seem to be mediated by calcium flux.

## 2. Results

### 2.1. In Vivo CXCR4 and ACKR3 Expression in Breast Cancer Tissue

**Figure 1:**
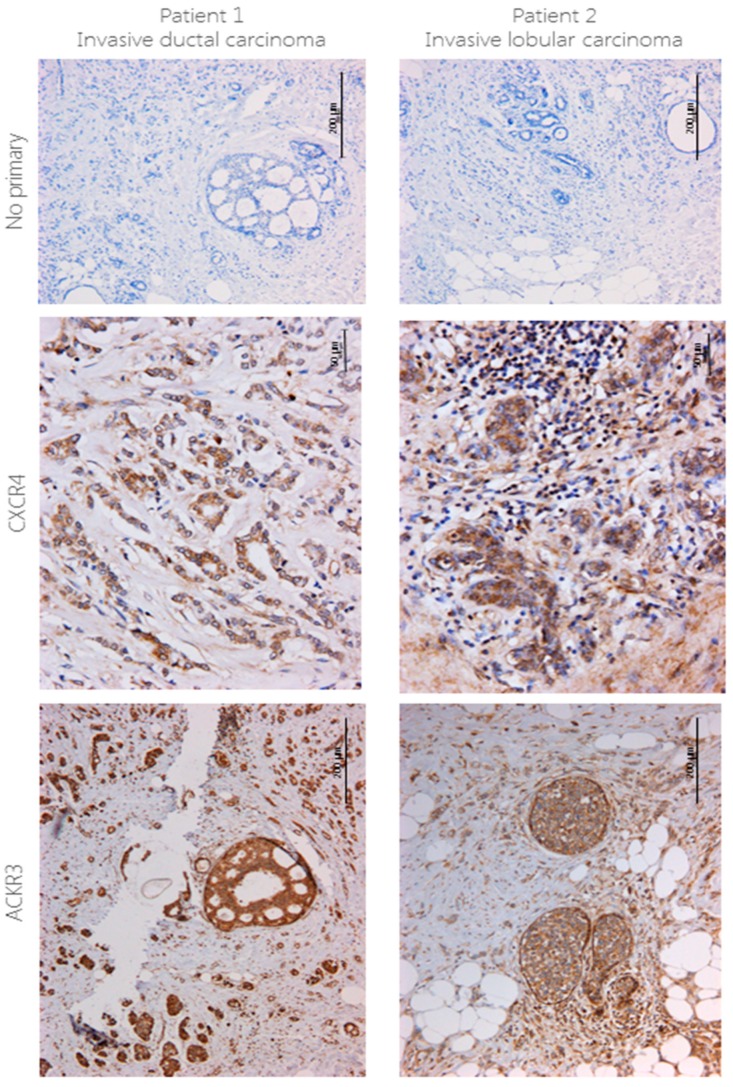
CXCR4 and ACKR3 staining using IHC in breast cancer tissue. 4 μm sections from human breast cancer were stained for CXCR4 (1:40) and ACKR3 (1:100) using immunohistochemistry following no pre-treatment or EDTA antigen retrieval pre-treatment, respectively. Briefly, protocol from the VECTASTAIN ABC HRP kit was followed, signal was developed using DAB and counterstained with haematoxylin. No primary antibody was used as a control. *n* = 2.

CXCR4 and ACKR3 expression was assessed in patient samples with invasive ductal carcinoma (Figure 1, left) and invasive lobular carcinoma (Figure 1, right) using immunohistochemistry. Expression of both receptors was high, with different staining patterns between cancer types. CXCR4 staining in ductal carcinoma was mainly present in the cytoplasm and cell surface, particularly around the ducts, whilst in lobular carcinoma staining was mostly cytoplasmic but was also present in the nucleus of most infiltrating cells. ACKR3 staining followed the same pattern as CXCR4—in ductal carcinoma ACKR3 was exclusively present in the cytoplasm and although it was also mostly cytoplasmic in lobular carcinoma, infiltrating cells also presented nuclei staining. Unlike CXCR4, however, staining was particularly strong in the walls of blood vessels. Positive and negative controls for ACKR3 staining can be found in Figure S1.

### 2.2. Expression of CXCR4 and ACKR3 on Transfected and Breast Cancer Cell Lines

**Figure 2:**
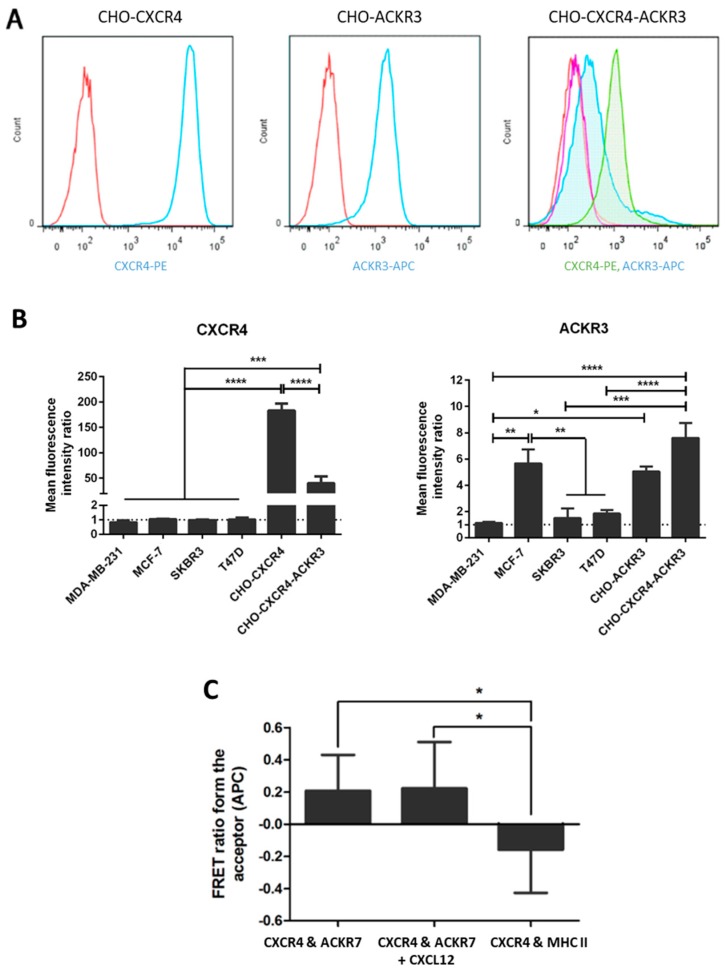
Expression of CXCR4 and/or ACKR3 in transfected CHO cells. CHO cells were transfected with pcDNA3.1/Zeo-CXCR4 and/or pcDNA3-ACKR3 using Effectene and selected with antibiotics. (**A**) Histograms showing CXCR4 or ACKR3 expression in the final selected colonies. Cells were stained with PE or APC-conjugated antibodies and expression was assessed using flow cytometry (Red = Isotype, blue = antibody) on CHO-CXCR4 (**left**) and CHO-ACKR3 (**middle**); and red = isotype for CXCR4, purple = isotype for ACKR3, green = CXCR4, blue = ACKR3 on CHO-CXCR4-ACKR3 (**right**) cells. (**B**) Mean fluorescence levels of the transfected CHO cells were determined and compared to several breast cancer cell lines to assess CXCR4 (**left**) and ACKR3 (**right**) receptor levels. Data represents the mean ± SEM of three independent experiments and statistical significance was calculated using a one way ANOVA (* *p* < 0.05). (**C**) Formation of heterodimers was assessed using fluorescence resonance energy transfer (FRET) and quantified through the FRET ratio from APC (the acceptor fluorochrome). Data represents the mean ± SEM of three independent experiments and statistical significance was calculated using a one way ANOVA (* *p* < 0.05, ** *p* < 0.01, *** *p* < 0.001, **** *p* < 0.0001).

qPCR and flow cytometric analyses were carried out to assess CXCR4 and ACKR3 expression in four breast cancer cell lines, however none expressed both receptors at high levels. Thus, to evaluate the roles of these receptors, CXCR4 and/or ACKR3 were stably overexpressed in CHO cells using lipid-based transfection and clones with highest expression levels at the cell membrane were selected and used in subsequent experiments (Figure 2A). Transient transfectants were used for CHO-CXCR4-ACKR3 cells and two different CHO-ACKR3 clones were assessed, however only one is shown in this study. Receptor expression of the transfected cells in comparison to breast cancer cell lines was evaluated using flow cytometric analyses (Figure 2B), showing that MDA-MB-231, MCF-7, SKBR3 and T47D express very low levels of CXCR4. On the other hand, MCF-7 cells expressed high levels of ACKR3 to a level comparable to CHO-CXCR4-ACKR3, whilst T47D expressed moderate levels and MDA-MB-231 and SKBR3 expressed very low levels. mRNA analysis produced similar results and confirmed the observed CXCR4 and ACKR3 expression.

In order to confirm whether CXCR4 and ACKR3 were forming heterodimers as previously reported [18,19,20], fluorescence resonance energy transfer (FRET) assays were carried out at saturating antibody concentrations. FRET analysis showed that CXCR4 and ACKR3 form heterodimers with or without the presence of CXCL12 (*p* < 0.05), with no FRET occurring with CXCR4 and MHC I (Figure 2C).

### 2.3. CXCR4 and ACKR3 Differentially Activate the Akt and ERK Oncogenic Pathways

Having engineered CHO cell lines overexpressing CXCR4, ACKR3, or both receptors, we compared their ability to activate ERK and Akt at different time points through Western Blot and cell-based ELISA. Since the ERK pathway is involved in migration [23,24] and Akt in proliferation [25,26], we assessed their phosphorylation after CXCL12 stimulation.

**Figure 3:**
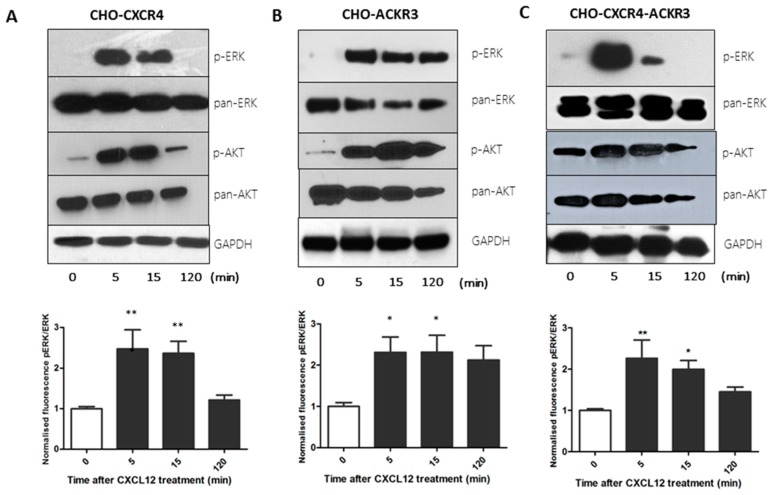
Western blot and cell-based ELISA show that CXCL12 treatment of transfected CHO cells differentially activates the ERK and AKT pathways. Serum starved (**A**) CHO-CXCR4, (**B**) CHO-ACKR3 and (**C**) CHO-CXCR4-ACKR3 cells were stimulated with 10 nM CXCL12 for 5, 15 and 120 min. (**Top**) Cells were lysed and immunoblots probed with p-ERK or p-Akt, stripped and re-probed for pan-ERK or pan-AKT and GAPDH as a loading control. Images are representative of three independent experiments. (**Bottom**) Cells were fixed with methanol and cell-based ELISA was performed as per protocol using p-ERK and total-ERK antibodies with fluorescence intensity readings at 600 and 450 nm. Data represents the mean ± SEM of three independent experiments and statistical significance was calculated using a one way ANOVA (* *p* < 0.05, ** *p* < 0.01).

CHO-CXCR4 cells presented early p-ERK activation that could be seen at 5 and 15 min but that had disappeared at 2 h and a similar pattern could be seen for p-Akt (Figure 3A). Conversely, a continuous activation for both p-ERK and p-Akt could be observed for up to 2 h in CHO-ACKR3 cells (Figure 3B). Interestingly, similarly to CHO-CXCR4, CHO-CXCR4-ACKR3 cells presented an intense ERK phosphorylation at 5 min that had disappeared at 2 h in both western blot and cell-based ELISA; but displayed constant p-Akt activation throughout two hours mirroring CHO-ACKR3 cells’ phosphorylation (Figure 3C).

### 2.4. ACKR3 Expression Can Alter CXCR4’s Internalization Pathway

**Figure 4:**
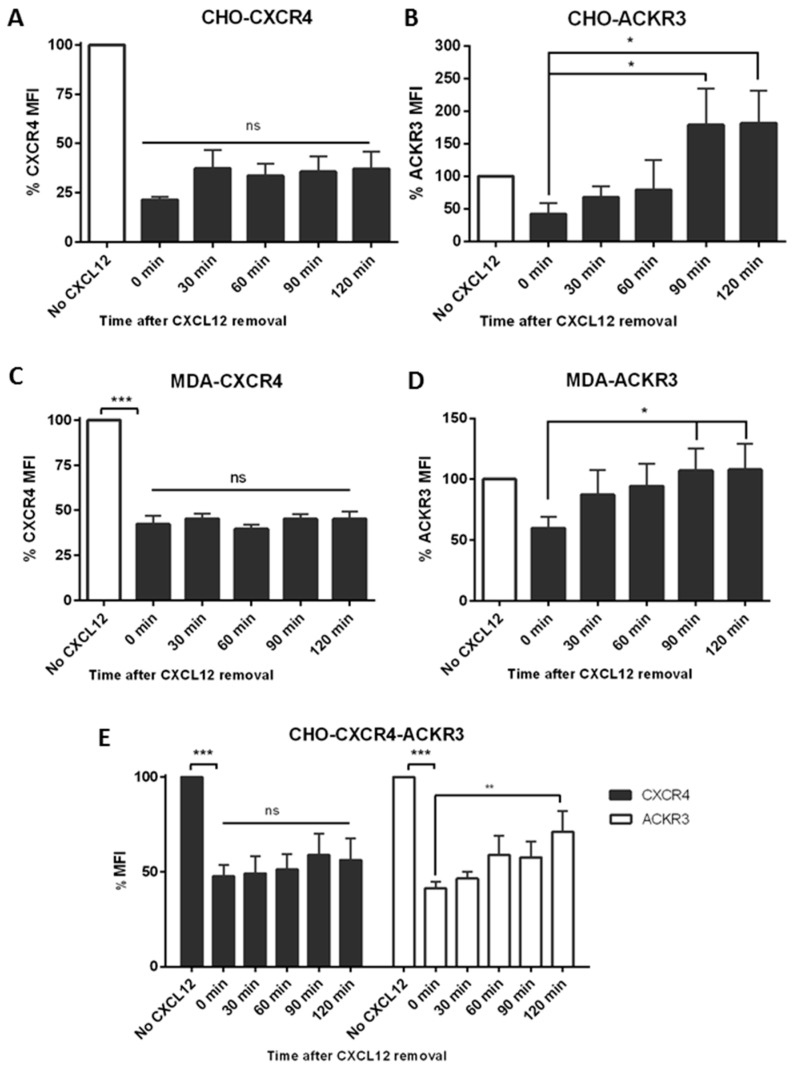
CXCR4 and ACKR3 follow different internalization pathways after CXCL12 stimulation. (**A**) CHO-CXCR4, (**B**) CHO-ACKR3, (**C**) MDA-MB-231-CXCR4, (**D**) MDA-MB-231-ACKR3 and (**E**) CHO-CXCR4-ACKR3 cells were treated with 10–50 nM CXCL12 for 15–30 min, then washed and incubated with chemokine-free media for up to 2 h to assess receptor recycling. Cells were labelled with CXCR4-PE and/or ACKR3-APC antibody and receptor’s mean fluorescence intensity (MFI) was measured using flow cytometry. Data represents the mean ± SEM of three independent experiments and statistical significance was calculated using a one way ANOVA (ns: not significant, * *p* < 0.05, ** *p* < 0.01, *** *p* < 0.001).

To investigate whether this difference in signalling was due to receptor internalization, cells were stimulated with CXCL12 before replacing the media and monitoring receptor levels using flow cytometry. An 80% reduction of CXCR4 levels could be observed after 30 min incubation with 10 nM CXCL12 in CHO-CXCR4 cells and when replaced with fresh media no recycling of the receptor could be seen after 2 h (Figure 4A)—the same pattern was observed in MDA-CXCR4 cells which were used as a physiological control (Figure 4C). Similarly, in CHO-ACKR3 a 50% receptor reduction could be seen after a 15-min incubation with 10 nM CXCL12 but when media was replaced ACKR3 expression was recovered after 60–90 min, with receptor levels increasing over the initial ones (Figure 4B) and correlating with the longer kinases’ activation. This phenomenon was also observed in MCF-7 but not in MDA-ACKR3 cells (Figure 4D), where ACKR3 just returned to original levels. In CHO-CXCR4-ACKR3, CXCR4 levels also remained at around 50% for up to two hours; conversely ACKR3 levels recycled back to the surface, although they did not reach the initial level after 2 h (Figure 4E). A representative histogram for the recycling after CXCL12 stimulation can be found in Figure S2.

**Figure 5:**
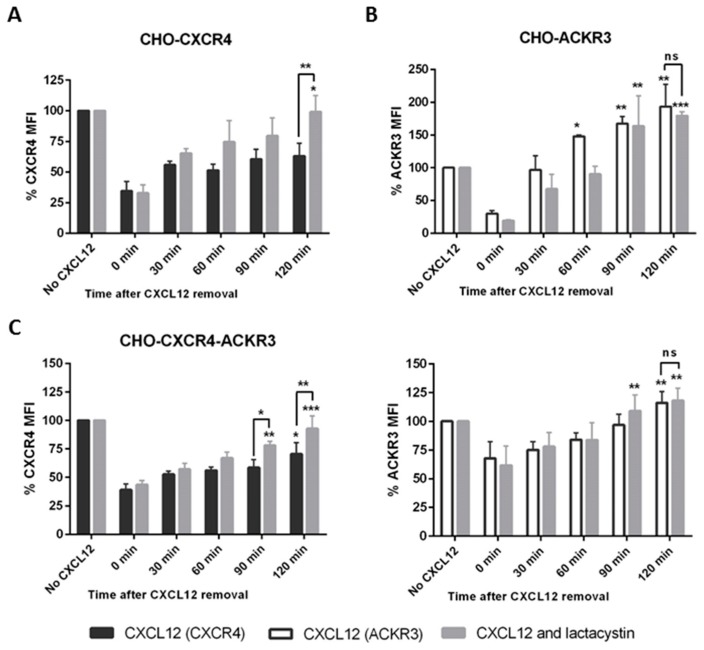
Preventing receptor degradation after CXCL12 stimulation using lactacystin, a proteasome inhibitor. (**A**) CHO-CXCR4, (**B**) CHO-ACKR3 and (**C**) CHO-CXCR4-ACKR3 cells were pre-treated for 1 h with 10 μM lactacystin (a proteosome inhibitor) before incubating with 10–50 nM CXCL12 for 15–30 min. Cells were then washed and incubated with chemokine-free media for up to 2 h before being labelled and analysed using a flow cytometer. Data represents the mean ± SEM of three independent experiments and statistical significance was calculated using a one way ANOVA (ns: not significant, * *p* < 0.05, ** *p* < 0.01, *** *p* < 0.001).

To confirm that CXCR4 was being degraded, cells were pre-treated with 10 μM of the proteasome inhibitor lactacystin for 1 h. Lactacystin prevented the degradation of CXCR4 in CHO-CXCR4 cells after CXCL12 stimulation, with receptor levels returning to initial levels 2 h after CXCL12 removal (Figure 5A). Pre-treatment with lactacystin had no effect in ACKR3 recycling in CHO-ACKR3 cells, confirming that ACKR3 was not degraded after internalization (Figure 5B). Furthermore, a similar pattern could be observed in CHO-CXCR4-ACKR3 cells, with CXCR4 expression returning to higher levels when pre-treated with lactacystin but having no effect in ACKR3 recycling (Figure 5C). A representative histogram for the recycling with and without lactacystin can be found in Figure S3.

### 2.5. ACKR3 Agonist VUF11207 Functions Differently to CXCL12 and Can Cross-Desensitize CXCR4

**Figure 6:**
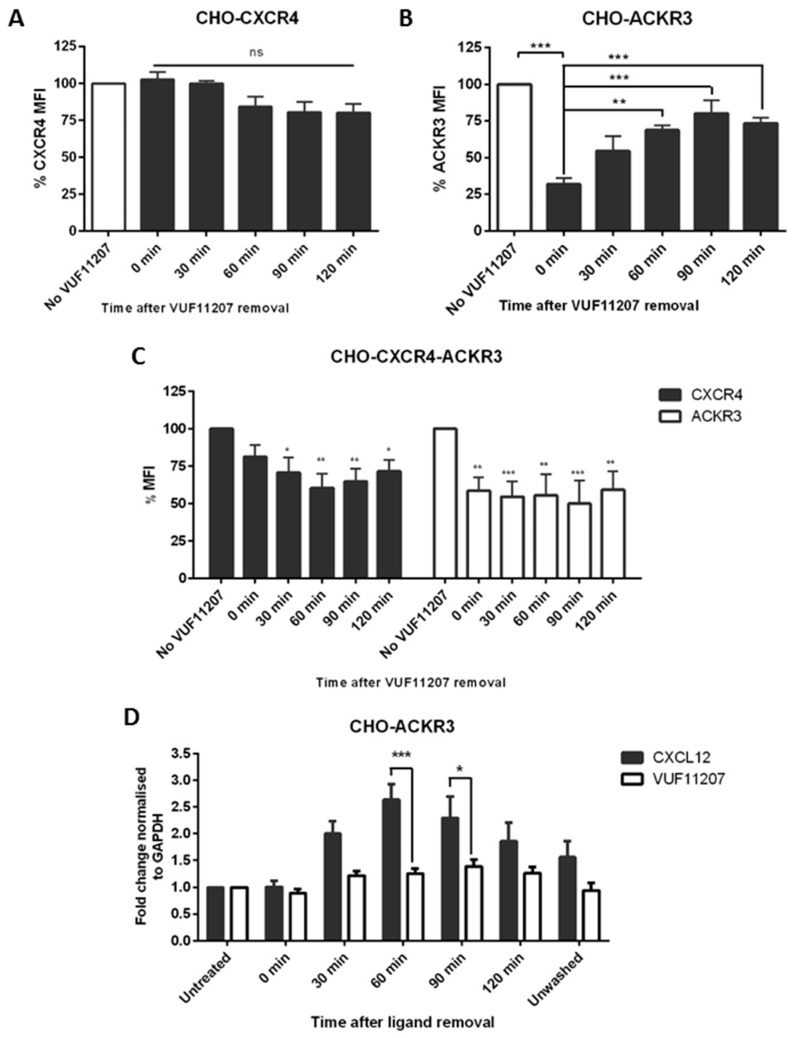
CXCR4 and ACKR3 follow different internalization pathways after VUF11207 stimulation. (**A**) CHO-CXCR4, (**B**) CHO-ACKR3 and (**C**) CHO-CXCR4-ACKR3 cells were stimulated with 1 nM VUF11207 for 30 min and then washed and incubated with agonist-free media for up to 2 h to assess receptor recycling. Cells were then labelled and receptor expression was measured using flow cytometry. Data represents the mean ± SEM of three independent experiments and statistical significance was calculated using a one way ANOVA (ns: not significant, * *p* < 0.05, ** *p* < 0.01, *** *p* < 0.001). (**D**) CXCL12 and VUF11207 have a different effect in ACKR3 transcription. CHO-ACKR3 cells were stimulated with 1 nM VUF11207 or 10 nM CXCL12 and recycling was assessed as described above. RNA was then extracted at each time point and ACKR3 expression was assessed using qPCR and normalized to GAPDH. Data represents the mean ± SEM of three independent experiments and statistical significance was calculated using a one-way ANOVA (* *p* < 0.05, *** *p* < 0.001).

After stimulation with 1 nM VUF11207 for 30 min, media containing the ligand was replaced by fresh complete media and receptor levels were monitored using flow cytometry. No significant effect in CXCR4 expression was seen in CHO-CXCR4 cells, confirming the specificity of the compound (Figure 6A). Similarly, to what was observed with CXCL12, ACKR3 expression in CHO-ACKR3 was recovered after the wash; however, it did not increase further than the original levels (Figure 6B)—the same trend was observed in MCF-7 cells. In CHO-CXCR4-ACKR3, CXCR4 levels decreased around 30%, whilst a 40–50% receptor reduction could be seen for ACKR3 (Figure 6C). Unlike with CXCL12, ACKR3 did not recycle back to the surface after VUF11207 stimulation.

In order to assess whether the increase in ACKR3 levels after CXC12 but not VUF11207-mediated recycling came from internal ACKR3 stored in vesicles or an increment in ACKR3 transcription, the receptor’s RNA levels were evaluated using qPCR. A significant increase in ACKR3 RNA expression could be observed at 60–90 min in the CXCL12-stimulated cells, whilst expression levels remained constant when cells had been treated with VUF11207 (Figure 6D). These results seem to suggest an upregulation triggered by the removal of CXCL12, with mRNA increase correlating with increased protein expression shortly thereafter. A representative histogram for the recycling after VUF11207 stimulation can be found in Figure S4.

### 2.6. ACKR3 Increases In Vitro Migration to CXCL12 in the Presence of CXCR4

**Figure 7:**
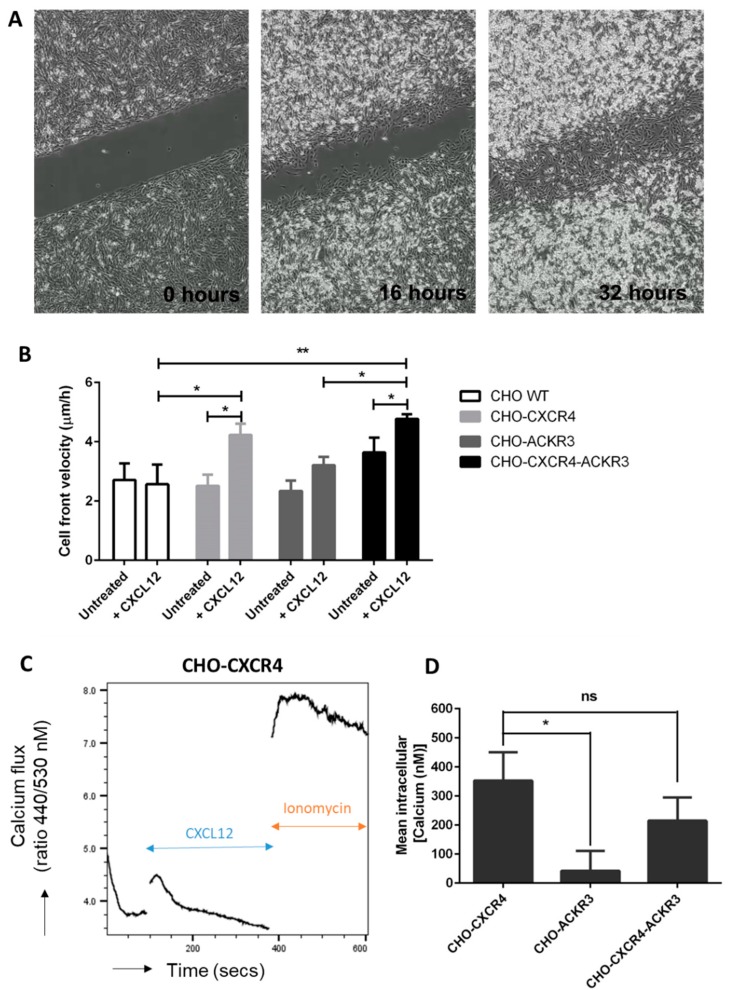
CXCR4 but not ACKR3, has an effect in wound healing after CXCL12 stimulation. CHO-WT, CHO-CXCR4, CHO-ACKR3 and CHO-CXCR4-ACKR3 cells were seeded into Ibidi inserts to create a “wound” 24 h later. Wells were then filled with serum-reduced media with or without 10 nM CXCL12 and wound closure was monitored for 48 h using the Nikon Biostation. (**A**) Example of images captured during a wound healing assay, magnification 4× (**B**) Cell front velocity was calculated from the wound area. Data represents the mean ± SEM of nine independent experiments and statistical significance was calculated using a two-way ANOVA with Bonferroni post-tests (* *p* < 0.05, ** *p* < 0.01). (**C**) CXCR4- but not ACKR3-expressing cells show calcium flux in response to CXCL12. Indo-1AM stained cells were stimulated with 10 nM CXCL12 followed by 4 μM ionomycin as a positive control and intracellular calcium flux was assessed. A representative plot showing calcium release in CHO-CXCR4 cells can be seen. (**D**) Ratio between free Indo-1 AM at 510 nm and calcium-bound Indo-1AM at 420 nm was calculated and concentration of calcium released was determined. Data is representative of three independent experiments and statistical significance was calculated using a one-way ANOVA (ns: not significant, * *p* < 0.05).

To evaluate the roles of CXCR4 and ACKR3 in CXCL12-induced migration in vitro, a scratch assay was performed (Figure 7A). Wound-healing assays revealed that CXCL12 treatment significantly increased cell front velocity in CHO-CXCR4 (4.229 μm/h) and CHO-CXCR4-ACKR3 (4.766 μm/h) in comparison to treated WT (2.565 μm/h), although to a lesser extent in the former. Addition of CXCL12 had no significant effect in CHO WT cells or CHO-ACKR3 cells, however wound speed closure was significantly decreased in CHO-ACKR3 cells in comparison to CHO-CXCR4-ACKR3 cells (Figure 7B). Proliferation assays were carried both in presence and absence of CXCL12 (10 nM). At 24, 48 & 72 h there was no significant change in cell proliferation between the wild type and transfectants (Figure S5).

### 2.7. Diminished Calcium Flux Induction in ACKR3 Expressing Cell Line

Since the discovery of calcium flux’s role in neutrophil’s migration [27], its role in many GPCR-mediated chemotaxis, including CXCR4, has been described [28]. We thus aimed to investigate whether the observed increases in cell front velocity were due to an increased migration or proliferation. We observed that both CHO-CXCR4 and CHO-CXCR4-ACKR3 cells successfully transduced intracellular calcium signals in response to CXCL12 (Figure 7C), with no statistically significant difference between the two. Calcium flux was however significantly reduced in CHO-ACKR3 cells compared to the CHO-CXCR4-ACKR3 cells (Figure 7D).

## 3. Discussion

Chemokines have been reported to regulate not only cancer cell migration and metastasis but also to stimulate tumour growth and invasion [6,11,29,30]. Since the discovery of ACKR3 as a CXCL12-binding chemokine receptor in addition to CXCR4 [31], its role in several cancers has been explored but its effects are still the source of many conflicting reports. For instance, ACKR3 has been reported to increase cell survival and adhesion [30]; yet studies suggests it both mediates [31,32] and does not affect chemotaxis [33] and its effects in conjunction with CXCR4 are even less clear [22,34,35]. Thus, a better understanding of the role of ACKR3, in particular in co-expression with CXCR4, is needed in order to further target these receptors. We separately observed that both ACKR3 and CXCR4 are overexpressed in breast cancer tumours of two patients with invasive ductal or lobular carcinoma, an increase also observed elsewhere [36,37]. However none of our breast cancer cell lines reflected this dual expression. Thus, CHO cells expressing one or both receptors were created and the presence of CXCR4/ACKR3 heterodimers was confirmed using FRET, a phenomena that has been previously described in literature [18,20,22].

After CXCL12 stimulation, we observed a transient p-ERK and p-AKT peak activation at 5 and 15 min in CHO-CXCR4 cells; conversely, p-ERK and p-AKT activation remained constant for up to 2 h in CHO-ACKR3 cells. These results suggest that ACKR3 expression and co-expression with CXCR4 can regulate CXCL12-driven phosphorylation of Akt but not ERK. This correlates with previous literature that suggests that ACKR3-mediated activation of these kinases is different from the canonical G-protein pathway in many ways. For instance, it has been reported that whilst G-protein-dependent ERK activation is quick and transient [38], β-arrestin-mediated activation is slower but more sustained [39,40]. Indeed, other studies also report that ACKR3 can activate both the ERK and Akt pathways through β-arrestin [41,42,43]. However kinetic reports vary, indicating that pathways may be activated differentially in time depending on the cell line. For instance, one study reports the complete opposite effect, with sustained ERK and transient Akt activation [44], whilst another reports a transient activation of both kinases [32], in partial agreement with our findings; and a third reports a sustained Akt activation in accordance with our observations [17]. Interestingly, p-ERK activation in CHO-CXCR4-ACKR3 showed a transient but intense peak at 5 min that disappeared with time, whilst Akt showed a sustained activation. This ERK pattern was also observed elsewhere [44,45,46] but sustained ERK activation has also been reported [22,47,48]. Once again, this is most likely due to the extreme variability in signalling pathways between cell lines, even within the same disease. Indeed, Heinrich, Lee [49] reported different activation patterns in two pancreatic cell lines which co-expressed ACKR3 and CXCR4.

We were interested to know whether these differences in ERK and Akt activation times could be correlated with the internalization of the receptor and whether it was then degraded or recycled back to the surface. Indeed, receptor internalization in transfected CHO cells has been described previously [31,50,51,52]. We observed that after CXCL12 stimulation, CXCR4 expression in CHO-CXCR4 and MDA-CXCR4 cells diminished and did not recover for up to 2 h, whilst ACKR3 levels in CHO-ACKR3 and MDA-ACKR3 had gone back to normal after 1 h. This is in accordance with other studies reporting that ACKR3 gets recycled after promoting CXCL12 degradation [14]. A similar pattern was seen in CHO-CXCR4-ACKR3 cells, albeit ACKR3 recovery levels were lower. This is in accordance to Uto-Konomi, McKibben [35], who reported that when ACKR3 and CXCR4 are co-expressed, CXCL12 stimulation induces degradation of CXCR4 but recycling of ACKR3. Co-internalization of both CXCR4 and ACKR3 was also reported in MCF-7 cells, although further degradation or recycling was not studied [45]. We also wanted to confirm that CXCR4 was being degraded through the proteasome pathway and indeed we observed CXCR4 recycling when cells were pre-treated with the proteasome inhibitor lactacystin. This is in accordance with other studies, which demonstrated that lactacystin could significantly increase CXCR4 levels [53,54].

Although numerous CXCR4 antagonists have been evaluated as therapeutic agents [55,56], ACKR3 therapies may provide a new anticancer avenue [57]. Indeed, ACKR3 antagonists have shown interesting results in inhibiting trans-endothelial migration [58] and ACKR3 agonists have also shown promise [35], proving that ACKR3 is a potential therapeutic target. Furthermore, desensitization through agonists has been shown to be a therapeutic avenue in anti-inflammatory diseases [59] and due to the formation of heterodimers, CXCR4 and ACKR3 are key candidates for cross-desensitization. In our study, it was observed that VUF11207 caused ACKR3 recycling in CHO-ACKR3 cells, albeit at lesser levels than when stimulated by CXCL12. qPCR results suggest that this difference in ACKR3 recovery levels could be partially explained by CXCL12-mediated induction of ACKR3 synthesis and not just by internal ACKR3 reserves [60]. Interestingly, VUF11207 stimulation of CHO-CXCR4-ACKR3 caused a reduction of both CXCR4 and ACKR3 and abrogated ACKR3 recycling, indicating that a different mechanism may be taking place after receptor activation with VUF11207. One reason for this difference could be that VUF11207 binds in a different pocket than CXCL12, causing changes in the recycling of the receptor [61]. Second, VUF11207 could be recycled back to the surface with the receptor, causing a continuous internalization. Indeed, after internalization CXCL12 is dissociated from the receptor in the late endosomes and degraded but some ligands remain bound [62], for instance AMD3100 has been reported to be bound to CXCR4 for over 24 h [63]. Third, due to their different molecular weight there may be changes in receptor dimerization after ligand binding—indeed, CXCL12 is approximately 8 kDa, whilst VUF11207 only has a molecular weight of 584 Da, which could result in different sterically hindrances that may cause an altered conformation [18].

ACKR3’s ability to mediate or enhance chemotaxis has been mixed, with some studies suggesting it plays a role in migration [22,31,32,34] and others reporting it does not [33,35,43,64,65]. Our findings are consistent with the latter, suggesting that ACKR3 alone does not mediate CXCL12-mediated chemotaxis but when co-expressed with CXCR4 it increases cell front velocity in the presence of CXCL12, similarly to that observed by Inaguma et al. [48]. These differences are most likely due to different expression levels of ACKR3, with higher levels scavenging all CXCL12 and impairing migration, whilst lower levels create a steep CXCL12 gradient that favours it. In contrast, CXCL12 significantly increased the wound healing speed in CHO-CXCR4 cells, a phenomena that has been reported in other wound healing assays [66,67].

Calcium flux has been widely reported to mediate cell migration through actin polarization, pseudopodia formation and adhesion to fibronectin [68] and thus we aimed to assess whether the differences observed in cell front velocity were due to an increased intracellular calcium release. Indeed, we observed CXCL12-mediated calcium flux induction in CHO-CXCR4 cells, a phenomena that has been described previously [30,69] and in correlation with previous studies [19,30,33] calcium flux was significantly impaired in CHO-ACKR3 cells. This further supports that ACKR3 does not interact with G-proteins due to having a DRYLSIT, instead of DRYLAIV, motif [70]. Interestingly, calcium flux in CHO-CXCR4-ACKR3 cells was not significantly different to CHO-CXCR4 or CHO-ACKR3, although its cell front velocity was significantly increased compared to CHO-ACKR3. This suggests that the difference observed in cell front velocity could be due to increased proliferation. However, we did not observe any significant change in proliferation both in the presence and absence of CXCL12. Other studies in endometrial cancer have shown that inhibition of these two receptors can reduce proliferation in cell lines [71] and both can be involved in proliferation in thyroid cancer, although their role depends on the cell line used [49]. Exposure to CXCL12 in prostate epithelial cell lines which express both receptors also causes an increased proliferative rate and blocking of CXCR4 or both receptors blocks CXCL12’s effect [72]. Interestingly, signalling through the Akt but not ERK pathway has been shown to induce proliferation in mammary epithelial cells [73], which correlates with the phosphorylation we observe in CHO-CXCR4-ACKR3 cells. Once again, the role of these kinases is likely cell line dependent [72].

In summary, our study highlights that CXCR4 and ACKR3 co-expression creates a distinct signalling entity with unique properties. Despite its over-expression in many cancer cell lines [30], the data presented suggests that ACKR3 does not have a role in chemotaxis but its presence can increase cell front velocity if co-expressed with CXCR4. Furthermore, we have seen that ACKR3 can induce ERK and Akt phosphorylation in a different kinetic and spatial sequence to CXCR4 and alter receptor internalization and thus may still be involved in other pathways such as NF-κB-mediated survival, tumour growth and angiogenesis. Furthermore, we have also shown that the ACKR3 agonist VUF11207 differentially desensitizes ACKR3, which could contribute to a new line of therapy.

## 4. Materials and Methods

### 4.1. Immunohistochemistry

Ethical approval for the use of paraffin-embedded samples from breast cancer tumours was submitted for proportionate review to the Research Ethics Committee (NHS Health Research Authority) and approved on the 14 March 2016 under the REC reference 16/YH/0117 (IRAS project ID 188839) by the Proportionate Review Sub-committee of the Yorkshire & The Humber—Leeds East. For the use of samples isolated prior to the Human Tissue Authority (HTA), approval had already been granted under the REC reference 06/Q0906/12. Informed consent was obtained from all subjects.

Immunohistochemistry of 4 μM paraffin-embedded cancer sections was carried out using the immunoperoxidase-based Vectastain ABC kit (Vector labs, Burlingame, CA, USA) as per the manufacturer’s instructions. Antigen retrieval was carried out using citrate buffer (pH 6) for 2 min and unspecific binding was blocked before anti-CXCR4 (1:40, MAB172, R&D systems, Minneapolis, MN, USA) or anti-ACKR3 (1:150, ab38089, Abcam, Cambridge, UK) antibodies were incubated with the sections for 2 h at room temperature. For anti-ACKR3 have used placenta as a positive control and non-immune rabbit serum as a negative control (Figure S4). Secondary and tertiary antibodies were added for 30 min before dispensing DAB on top of the samples (DAB Peroxidase (HRP) Substrate Kit, Vector labs, Burlingame, CA, USA) and counterstaining with haematoxylin.

### 4.2. Cell Culture

Wild-type CHO cells (CHO WT) (ATCC) were cultured in Ham’s F12 medium (Thermo Fisher Scientific, Waltham, MA USA) supplemented with 10% FBS, 100 U/mL penicillin, 100 μg/mL streptomycin and 0.146 g/L L-glutamine in a humidified incubator with 5% CO_2_ at 37 °C. MDA-MB-231-CXCR4 and MDA-MB-231-ACKR3 were a gift from Kathryn E Luker, Ph.D (Centre for Molecular Imaging, Department of Radiology, University of Michigan, Ann Arbor, MI, USA) and were grown as previously described [14].

### 4.3. Construction of Stable and Transient Transfectants

The mammalian cell expression vector pcDNA3.1Zeo (Thermo Fisher Scientific, Waltham, MA, USA) containing CXCR4 was transfected into CHO WT cells as described previously [74]. ACKR3 cDNA from the pCMV-XL5/ACKR3 vector (OriGene, Rockville, MD, USA) was cut and cloned into a pcDNA3 vector and transfected into CHO WT and CHO-CXCR4 cells using Effectene (Qiagen, Hilden, Germany). Stable CHO-ACKR3 cell lines were obtained by growing transfected cells at low density for 24 h followed by the addition of 800 μg/mL G418 for selection over 3 weeks. Clones were picked and expanded before ACKR3 expression assessment and a single cell dilution of the highest expressing colonies was carried out. Transient CHO-CXCR4-ACKR3 transfected cells were created using Effectene (Qiagen, Hilden, Germany) 24 h prior to the assay.

### 4.4. Cell Surface Expression of Chemokine Receptors

The CXCR4-Phycoerythrin (PE) antibody (Clone 12G5, R&D systems, Minneapolis, MN, USA) and ACKR3-Allophycocyanin (APC) antibody (Clone 11G8, R&D systems, Minneapolis, MN, USA) used in this study were all fully optimized before the collection of quantitative data. Briefly, 2 × 10^5^ cells were incubated with the corresponding antibody in 50 μL FACS buffer (2% BSA/PBS) for 30 min or 90 min, respectively, at 4 °C before washing and resuspending cells in 100 μL FACS buffer. Receptor expression was recorded using a FACS Canto II (BD Biosciences, Franklin Lakes, NJ, USA) and analysed using FlowJo 7 (FlowJo, Ashland, OR, USA).

To assess receptor internalization, cells were serum starved for one hour before being stimulated with 10 nM CXCL12 (for CHO-CXCR4, CHO-ACKR3, MDA-CXCR4 and MDA-ACKR3) or 50 nM CXCL12 (for CHO-CXCR4-ACKR3) for 15 min (CHO-ACKR3) or 30 min (CHO-CXCR4, MDA-CXCR4, MDA-ACKR3 and CHO-CXCR4-ACKR3). Chemokine was then washed with PBS and cells were incubated with fresh media at 37 °C for various time periods before harvesting. If necessary, cells were pre-incubated with 10 μM lactacystin for 1 h. For VUF11207, all cells were incubated 1 nM VUF11207 for 30 min. Cells were then labelled and analysed by FACS as described above.

### 4.5. FRET

FRET was assessed by flow cytometry with excitation of CXCR4-PE at 488 nm and measurement of emission from ACKR3-APC at 675 nm [75]. To demonstrate that FRET was non-random, both antibodies were titrated to a saturating concentration and MHC I-APC (2G5, Novus Biologicals, Centennial, CO, USA) was used as a negative control. Energy transfer efficiencies calculated as the ratio between APC’s mean fluorescence intensity (MFI) on its own and with CXCR4.

### 4.6. Western Blot

After serum starvation for one hour, cells were stimulated with 10 nM CXCL12 for various time periods before being lysed with 50 μL of CelLytic M Cell Lysis Reagent (Sigma-Aldrich, St. Louis, MO, USA) supplemented with cOmplete™ Mini Protease Inhibitor Cocktail (Sigma-Aldrich, St. Louis, MO, USA) and PhosSTOP™ Phosphatase Inhibitor Cocktail (Sigma-Aldrich, St. Louis, MO, USA). Cell lysates were run in a 10% SDS-PAGE gel electrophoresis and then transferred onto nitrocellulose membranes. Membrane was blocked with 5% BSA/PBS for one hour before being incubated with p-ERK (1:1000, Cell Signalling, Danvers, MA, USA) or p-Akt (1:1000, Cell Signalling, Danvers, MA, USA) antibodies overnight and detected using a horseradish peroxidase-linked secondary antibody (1:4000, Santa Cruz Biotechnology, Dallas, TX, USA). Immunoblots were developed using SuperSignal™ West Pico Chemiluminescent Substrate (ThermoFisher, Waltham, MA, USA) according to manufacturer’s instructions. Re-blotting was performed to quantify the total amount of ERK (1:2000, Cell Signalling, Danvers, MA, USA), Akt (1:2000, Cell Signalling, Danvers, MA, USA) and GAPDH (1:2000, Abcam, Cambridge, UK) for normalization.

### 4.7. Cell-Based ELISA

Relative ERK phosphorylation of transfected CHO cells was determined using a cell-based ELISA (R&D systems, Minneapolis, MN, USA) as per manufacturer’s instructions. Briefly, cells were grown in 96-well plates and stimulated with 10 nM CXCL12 for various time periods before being fixed. Endogenous peroxidase was quenched and cells were blocked and incubated with p-ERK and total ERK antibodies overnight. The next day, cells were incubated with secondary antibodies conjugated to either HRP or alkaline phosphatase (AP) and fluorescence was measured at two different wavelengths.

### 4.8. qPCR

mRNA expression of ACKR3 after treatment with CXCL12 or VUF11207 was assessed using the StepOnePlus^TM^ PCR machine (Applied Biosystems Waltham, MA, USA). Cells were treated with either ligand as described above and lysed using the RNeasy Mini Kit (Qiagen, Hilden, Germany). 1 μg RNA was used for reverse transcription with the Tetro cDNA synthesis kit (Bioline, London, UK) and cDNA was used for qPCR with the SensiFast Probe Hi-ROX Mix (Bioline, London, UK) and ACKR3 Taqman probes (Applied Biosystems Waltham, MA, USA). RNA expression was normalized to GAPDH and quantified using the 2^−ΔΔCt^ method.

### 4.9. Wound Healing Assay

In order to assess cell migration through time, a wound healing assay was carried out using 2-well Ibidi inserts (Ibidi, Martinsried, Germany). Briefly, inserts were placed into a 12-well plate and 70 μL of 4 × 10^5^ cells/mL were added to each side of the insert and left to grow into a confluent monolayer under serum starvation conditions. The next day, the insert was removed to create the “scratch” and serum-reduced media with or without 10 nM CXCL12 was added. Wound closure was then monitored for 48 h using the NIKON Biostation CT (Nikon, Tokyo, Japan) and analysed using the NIS-Elements AR software (Nikon, Tokyo, Japan). Cell front velocity was calculated as described in Ibidi’s website (Available online: https://ibidi.com/img/cms/support/AN/AN21_Wound_Healing_Assay.pdf).

### 4.10. Calcium Flux

Changes in intracellular Ca^2+^ concentrations in response to 10 nM CXCL12 and 1 µM of the calcium ionophore ionomycin (Sigma-Aldrich, St. Louis, MO, USA) were analysed using calcium-responsive dye fluorescence and flow cytometry. Briefly, 1 × 10^6^ cells were stained with 4 µL of 1 mg/mL Indo-1 AM in 100 µL supplemented PBS (containing 1 mmol/L CaCl_2_, 1 mmol/L MgCl_2_ and 1% FBS) for 30 min at 37 °C. Cells were washed and resuspended in 1 mL supplemented PBS and rested at 37 °C for 30 min before analysis. The sample was then analysed using the Fortessa X-20 flow cytometer BD Biosciences, Franklin Lakes, NJ, USA) with the UV laser for excitation, with 450/50 and 530/30 nm emissions recorded. Baseline readings were taken for 1 min before the addition of 10 nM CXCL12 for 4 min, followed by 1 µM ionomycin. Finally, the ratio between the two wavelengths was calculated and plotted against time in order to calculate the peaks of calcium release. To calculate the intracellular calcium concentration, the following formula was used: [Calcium (nM)] = Kd × ((CXCL12 peak − baseline peak)/(Ionomycin peak − CXCL12 peak)), where Kd is 844 nM, the dissociation constant of calcium and Indo-1 AM [76,77].

### 4.11. Statistical Analyses

All results are expressed as means ± SEM of replicate samples. The significance of changes was assessed by the application of an ANOVA with Bonferroni post-test or two-tailed Student’s *t*-test. All data were analysed using Prism 5.0 software (GraphPad Software, La Jolla, CA, USA).

## Supplementary Materials

Supplementary materials can be found at http://www.mdpi.com/1422-0067/19/11/3592/s1.
