# Correction: del Molino del Barrio et al. Breast Cancer: An Examination of the Potential of ACKR3 to Modify the Response of CXCR4 to CXCL12. *Int. J. Mol. Sci.* 2018, *19*, 3592

**DOI:** 10.3390/ijms242317108

**Published:** 2023-12-04

**Authors:** Irene del Molino del Barrio, Georgina C. Wilkins, Annette Meeson, Simi Ali, John A. Kirby

**Affiliations:** 1 Applied Immunobiology and Transplantation Group, Institute of Cellular Medicine, Medical School, University of Newcastle upon Tyne, Newcastle upon Tyne NE2 4HH, UK; 2 Institute of Genetic Medicine, International Centre for Life, University of Newcastle upon Tyne, Newcastle upon Tyne NE1 3BZ, UK

The authors and Editorial Office were made aware of an error in a figure within the original publication [1].

During the compilation of the images for this article, an error occurred whereby the incorrect images for Figure 1, “no primary” antibody control for Patient 1 and 2, were published. The authors provided the correct original image files to the Editorial Office, and Figure 1 has now been updated with the correct “no primary” Patient 1 and 2 images. The magnification is different for the Patient 1 and 2 no primary controls. The authors state that the scientific conclusions are unaffected. This correction process was supervised and approved by the Academic Editor. 

## Figures and Tables

**Figure 1:**
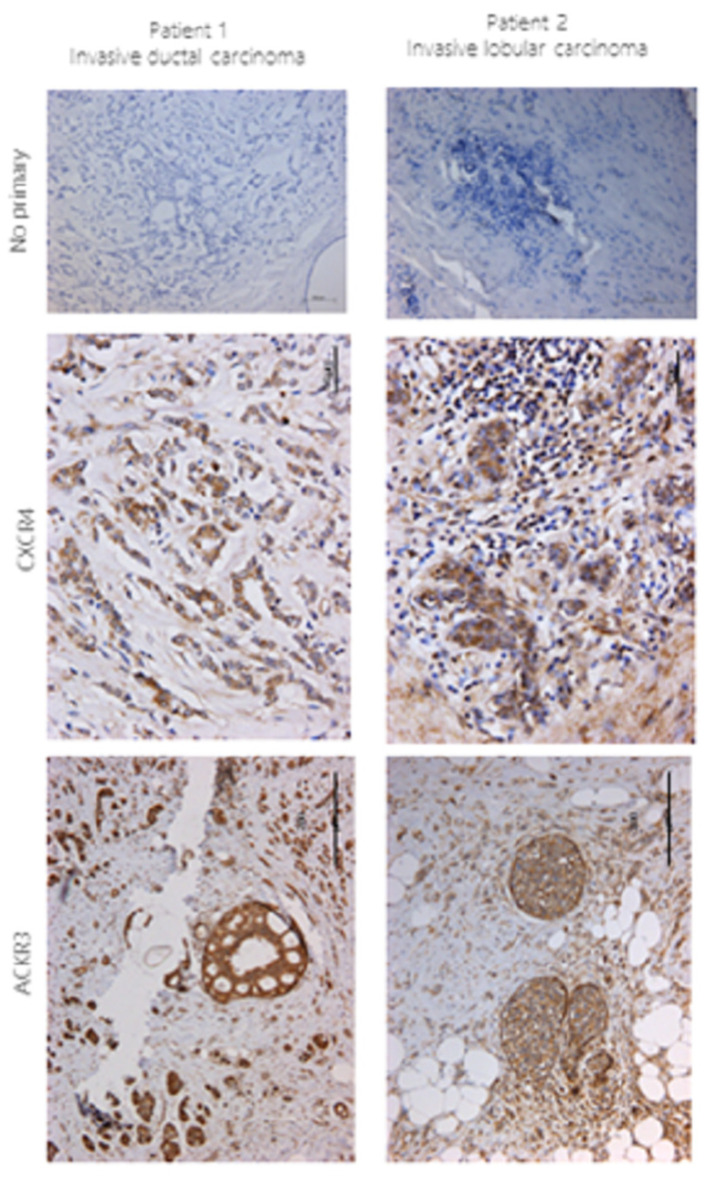
CXCR4 and ACKR3 staining using IHC in breast cancer tissue. Sections 4 μm in size from human breast cancer were stained for CXCR4 (1:40) and ACKR3 (1:100) using immunohistochemistry following no pre-treatment or EDTA antigen retrieval pre-treatment, respectively. Briefly, the protocol from the VECTASTAIN ABC HRP kit was followed; the signal was developed using DAB and counterstained with haematoxylin. No primary antibody was used as a control. n = 2, patient 1 control, scale bar = 100 μm; patient 2 control, scale bar = 200 μm.

## References

[1] del Molino del Barrio I., Wilkins G.C., Meeson A., Ali S., Kirby J.A. (2018). Breast Cancer: An Examination of the Potential of ACKR3 to Modify the Response of CXCR4 to CXCL12. Int. J. Mol. Sci..

