# Supplementary material for: Breast Cancer: An Examination of the Potential of ACKR3 to Modify the Response of CXCR4 to CXCL12
Source: Int J Mol Sci. 2018 Nov 14;19(11):3592. doi: 10.3390/ijms19113592 (PMC6274818; doi:10.3390/ijms19113592)
Supplement: Supplementary file 1 [file ijms-19-03592-s001.zip › ijms-365067-SI.pdf]

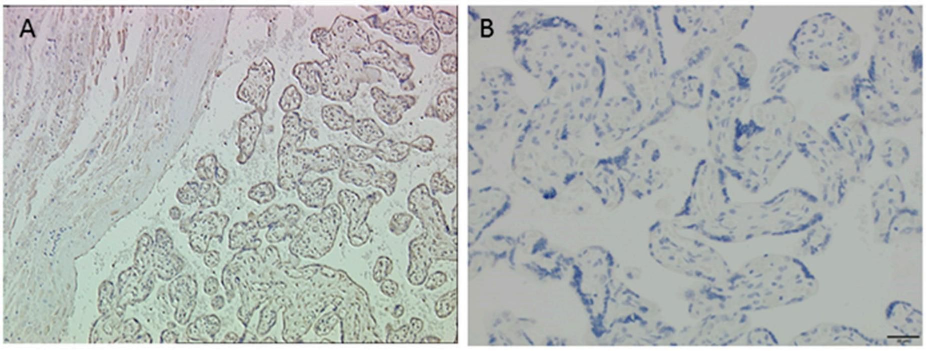

Figure S1. Controls for ACKR3. 4 μm sections from human placenta were stained for (**A**) ACKR3 (1:100) or (**B**) incubated with pre-immune rabbit serum using immunohistochemistry following antigen retrieval pre-treatment (EDTA antigen retrieval). Briefly, protocol from the VECTASTAIN ABC HRP kit was followed, signal was developed using DAB (brown stain) and counterstained with haematoxylin (blue).

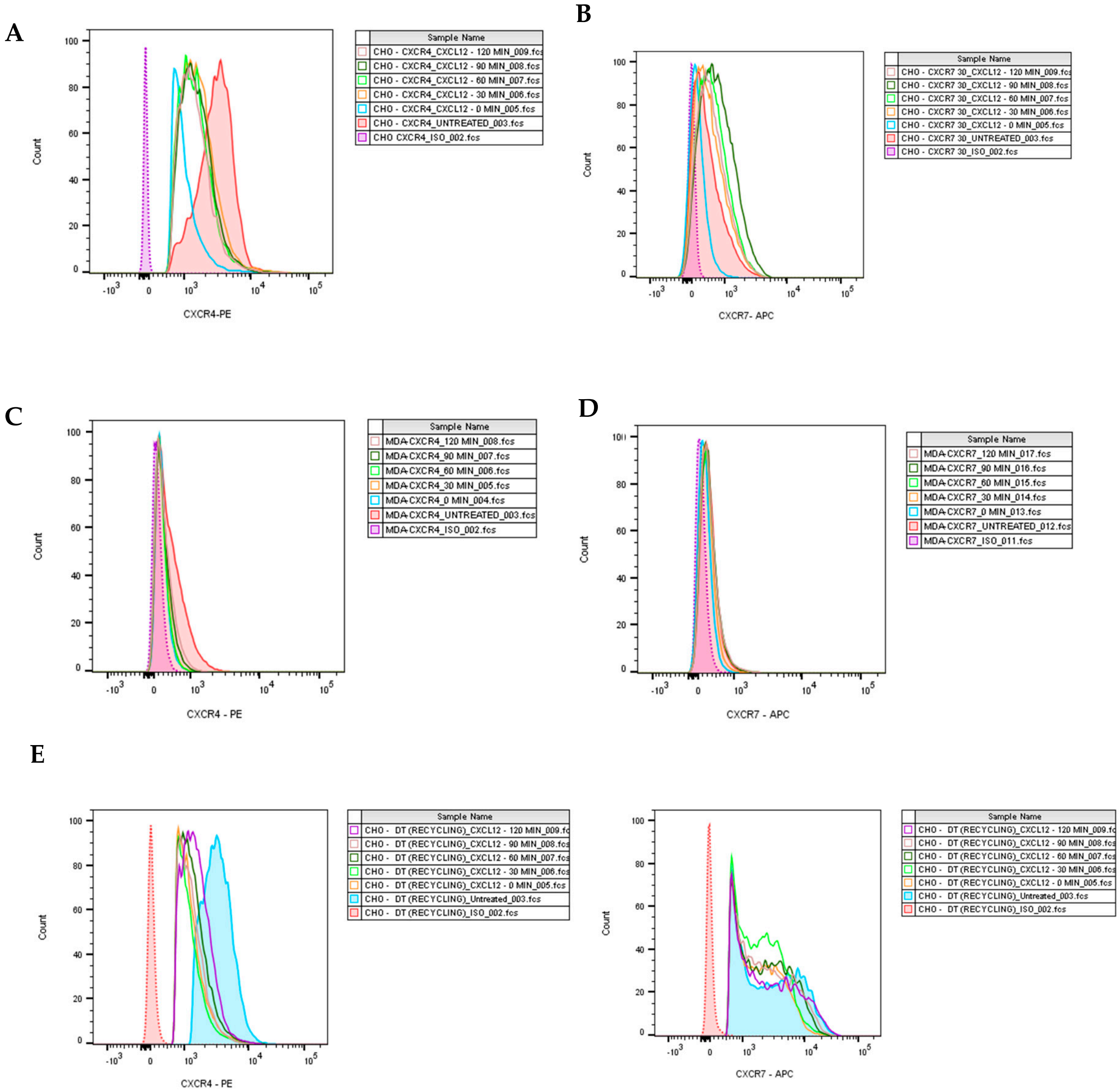

Figure S2. CXCR4 and ACKR3 follow different internalization pathways after CXCL12 stimulation. (**A**) CHO-CXCR4, (**B**) CHO-ACKR3, (**C**) MDA-MB-231-CXCR4, (**D**) MDA-MB-231-ACKR3 and (**E**) CHO-CXCR4-ACKR3 cells were treated with 10–50 nM CXCL12 for 15–30 min, then washed and incubated with chemokine-free media for up to 2 h to assess receptor recycling. Cells were labelled with CXCR4-PE and/or ACKR3-APC antibody and representative histograms of the staining are depicted.

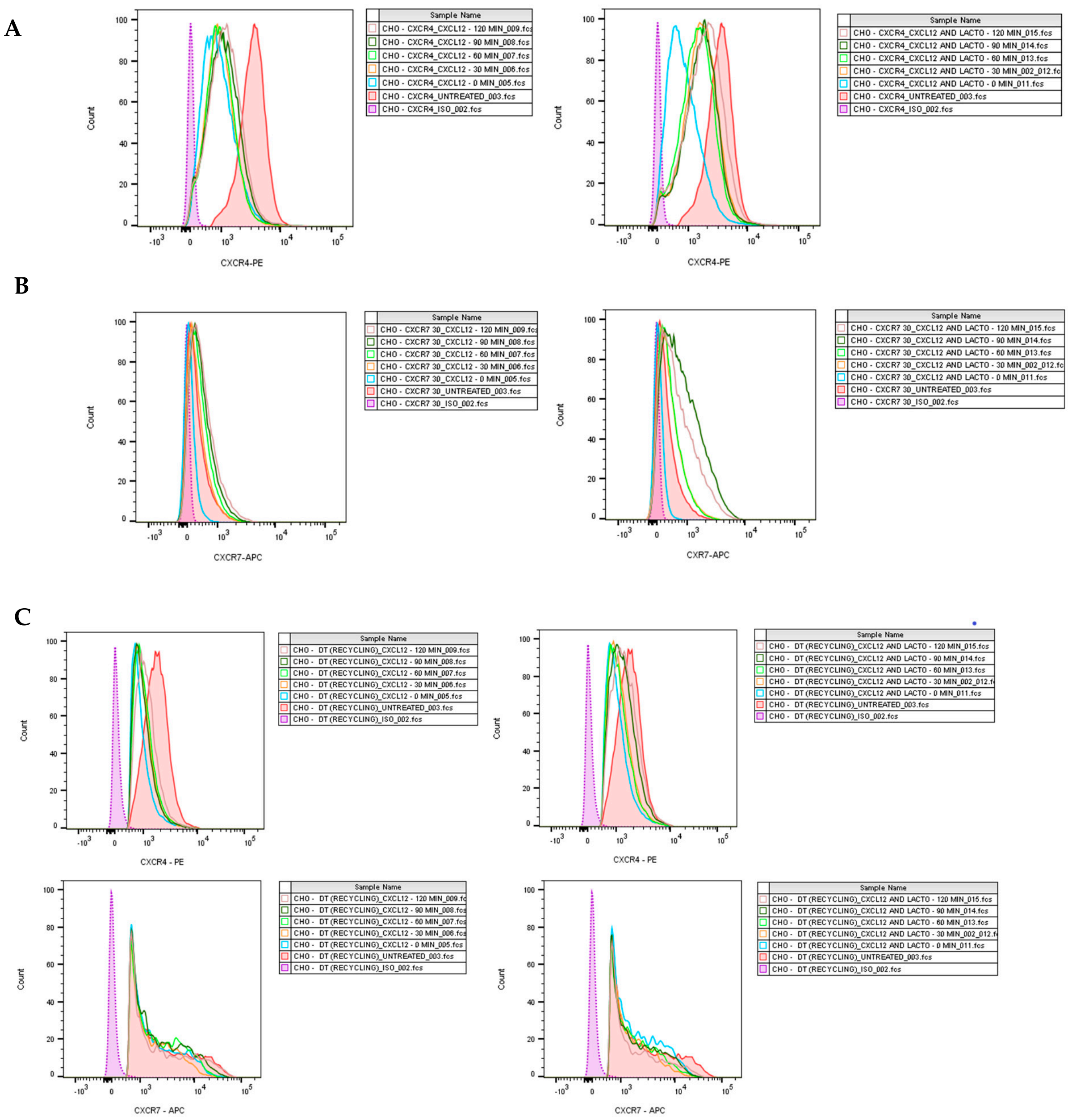

Figure S3. Preventing receptor degradation after CXCL12 stimulation using lactacystin, a proteasome inhibitor. (**A**) CHO-CXCR4, (**B**) CHO-ACKR3 and (**C**) CHO-CXCR4-ACKR3 cells were pre-treated for 1 h with 10 µM lactacystin (a proteosome inhibitor) before incubating with 10–50 nM CXCL12 for 15–30 min. Cells were then washed and incubated with chemokine-free media for up to 2 h before being labelled with CXCR4-PE and/or ACKR3-APC antibody. Cells were labelled with CXCR4-PE and/or ACKR3-APC antibody and representative histograms of the staining are depicted.

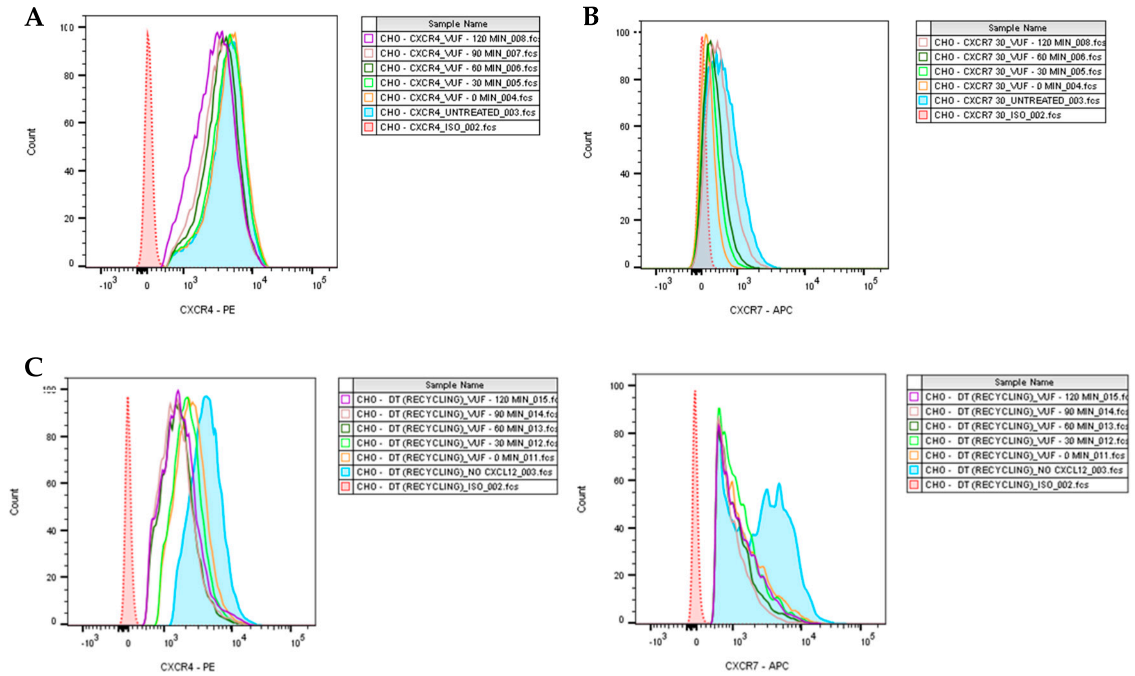

Figure S4. CXCR4 and ACKR3 follow different internalization pathways after VUF11207 stimulation. (**A**) CHO-CXCR4, (**B**) CHO-ACKR3 and (**C**) CHO-CXCR4-ACKR3 cells were stimulated with 1 nM VUF11207 for 30 min and then washed and incubated with agonist-free media for up to 2 h to assess receptor recycling. Cells were then labelled and receptor expression was measured using flow cytometry.

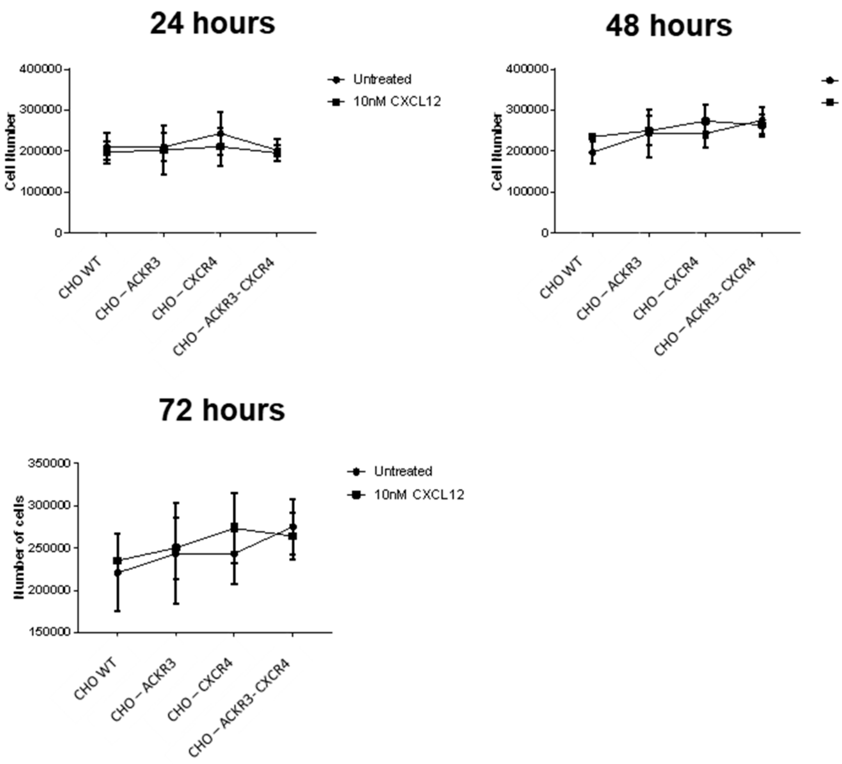

Figure S5. Proliferation of cells in presence and absence of 10 nm CXCL12. CHO WT, CHO-ACKR3, CHO-CXCR4 and CHO-ACKR3-CXCR4 transfectants were seeded in duplicate in a 24-well plate. After four hours, the cells were treated with 10 nM CXCL12 or were left untreated. At 24, 48 or 72 h following CXCL12 stimulation, cells were washed in PBS and detached with Accutase®. The cells were counted using a haemocytometer and analysed using Graphpad Prism. *n* = 3.
